# Application of inulin in bread: A review of technological properties and factors affecting its stability

**DOI:** 10.1002/fsn3.3141

**Published:** 2022-11-14

**Authors:** Faezeh Mohammadi, Ameneh Shiri, Sima Tahmouzi, Neda Mollakhalili‐Meybodi, Amene Nematollahi

**Affiliations:** ^1^ Department of Food Sciences and Technology School of Public Health, Shahid Sadoughi University of Medical Sciences Yazd Iran; ^2^ Research Center for Food Hygiene and Safety Shahid Sadoughi University of Medical Sciences Yazd Iran; ^3^ Department of Food Safety and Hygiene School of Health, Fasa University of Medical Sciences Fasa Iran

**Keywords:** bread, inulin, prebiotics, stability, technological characteristics

## Abstract

Due to its dual function, inulin is an important prebiotic compound in the cereal industry, especially in bread production. In other words, improving technological features and creating health properties (such as reducing the risk of type 2 diabetes, heart disease, metabolic syndrome, and osteoporosis) have led to the widespread use of this compound. Inulin has many important technological functions in bread, including its ability to interact with water, create structure, and influence rheological properties, texture, and overall acceptability of the final product. Nevertheless, bread processing conditions can influence the structural integrity of inulin and thus affect its technological efficiency. Therefore, this review article aims to investigate the technological properties and factors affecting the stability of inulin during bread processing conditions. Generally, the addition of inulin could considerably improve the technical performance of bread. However, the stability of inulin depends on the formulation components, type of fermentation, and baking process.

## Graphical abstract

This article investigated the different function of inulin in bread. Effects of adding inulin in bread technical properties are investigated. Effect of different factors on the stability of inulin during bread processing conditions has been investigated.

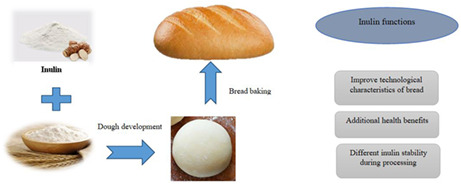

## 1 INTRODUCTION

Bread is one of the essential components of the diet, which is mainly made from wheat flour (Mohammadi et al., 2021). Gluten protein is responsible for the unique properties of wheat dough (Meybodi et al., 2019), which constitutes more than 80% of wheat proteins. It consists of two proteins, gliadin and glutenin, which play a critical role in producing the viscosity and elasticity of the dough. Gluten can also affect some factors such as water absorption, dough mixing time, the storage capacity of gas (produced during processing), and ultimately the quality of bread (Natalia Drabińska et al., 2016).

There are some challenges regarding the production and consumption of some bakery products, especially wheat bread. Although the use of whole‐wheat flour improves the nutritional properties of the finished bread due to its high content of fiber and antioxidant compounds, there are several problems with this type of flour. Some of these vulnerabilities include nutritional issues such as reduced mineral bioavailability (due to phytic acid and insoluble fibers) and technological problems, including reduced specific volume, moisture, and water activity as increased hardness (Boita et al., 2016). Therefore, white wheat flour (containing 21% bran) is recommended to produce bulk wheat bread (Dastmalchi & Razavi, 2016). In addition, wheat flour‐based baked goods are also prohibited for people with celiac disease and other gluten‐related disorders. Despite switching to a gluten‐free diet such as gluten‐free bread, it is impossible to benefit from the desired technological features of these products (Horstmann et al., 2019). Furthermore, there is also a need to freeze dough as it is a convenient and cost‐effective way to meet the nutritional needs of people who are pressed for time and do not have access to various food sources such as travelers, mountaineers, astronauts, and soldiers (Rahman, 2007). However, this procedure is associated with adverse changes in the technological characteristics of wheat bread, including a reduction in specific volume, increased fermentation time, and a weakening of the gluten network structure (Ke et al., 2020). Hence, it is challenging to fortify white wheat bread and simulate gluten structure in gluten‐free bread.

In recent years, dietary fiber has attracted much attention because of its beneficial effects on health, including improving gastrointestinal health and preventing constipation, cardiovascular disease, and cancer (Chen et al., 1988). These compounds are divided into two categories depending on their solubility in water: “soluble dietary fiber” and “insoluble dietary fiber” (Sciarini et al., 2017). It is reported that the addition of insoluble dietary fiber negatively affects the technological properties of the final product (Arp et al., 2020). It may also increase the postprandial glucose level of fortified food formulation by providing a physical barrier and preventing structure formation. Soluble fiber, on the other hand, can increase lipid and carbohydrate metabolism and lower cholesterol and blood glucose levels after eating (Kalyani Nair et al., 2010).

Prebiotics are indigestible and soluble dietary fibers that are resistant to gastric acid and hydrolysis by mammalian enzymes. In the digestive tract, they could selectively stimulate certain probiotic bacteria. Consequently, prebiotics may reduce the risk of type 2 diabetes, metabolic syndrome, osteoporosis, inflammatory bowel disease, irritable bowel syndrome, obesity, and dysentery (A Parnell & A Reimer, 2012). One of the most important prebiotic compounds with wide application in the bakery industry is inulin, which, in addition to functional and desirable effects on the technological properties of bread, has several beneficial effects on health (Glibowski & Bukowska, 2011).

Considering the importance of wheat bread in human nutrition and the need for its enrichment and mimicking of the three‐dimensional network of gluten in some applications, the inclusion of inulin as soluble dietary fibers needs to be studied. Since the technological properties of bread, which are crucial for consumer acceptance, are highly influenced by the formulation, this study aims to review the effects of inulin on the technological properties of bread. Furthermore, since inulin's health‐promoting effects are attributed to its regular and specific consumption in intact form, the factors affecting its stability during bread processing will be investigated.

## 2 INULIN: CHEMICAL AND HEALTH PROPERTIES

**FIGURE 1:**
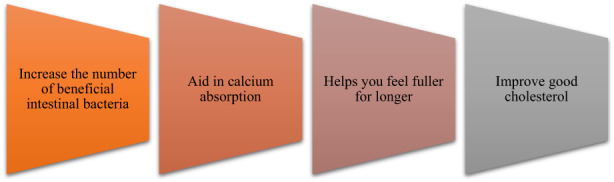
Benefits of using inulin

Inulin is a water‐soluble storage polysaccharide composed of fructose units linked by 1: 2 beta bonds with a degree of polymerization (DP) of 2–60 (Shoaib et al., 2016). Figure. 1 illustrates its benefits. Although the main extraction source of inulin is chicory root (Wang et al., 2002), it can be found in more than 36,000 plant species such as bananas, onions, artichokes, asparagus, leeks, chicory root, garlic, and pickled potatoes. There are three categories of inulin, including short chains (DP < 10), long chains (DP > 23), and natural (DP = 2–60) types. Short‐chain inulin has higher solubility and sweetness than the long‐chain type and is used as a sugar substitute and mouthfeel enhancer in low‐calorie products. Long‐chain inulin, on the other hand, is used as a filler and fat substitute because it is less soluble and more viscous (Natalia Drabińska et al., 2016). The inulin compound is colorless and tastes neutral. Although it has little effect on the taste of the finished product, it can significantly affect the organoleptic properties of this product in terms of DP (Kalyani Nair et al., 2010).

The β‐configuration of inulin prevents it from being hydrolyzed by enzymes in the upper gastrointestinal tract of humans, allowing it to enter the large intestine intact and be selectively fermented by beneficial intestinal bacteria. According to this property, inulin belongs to the category of prebiotics and low‐calorie dietary fibers (compared to other carbohydrates) (Morreale et al., 2019). This compound can also reduce the glycemic response, enhance gastrointestinal health, affect fat metabolism, and reduce the risk of osteoporosis and colon cancer (Kalyani Nair et al., 2010). The use of artichoke extract (as a source of inulin) in rat's drinking water has been shown to lower total cholesterol and low‐density lipoprotein (LDL) and increase high‐density lipoprotein (HDL) levels (Naci et al., 2019). Considering the functional benefits for celiac patients, the addition of 12% inulin‐type fructans (ITFs) to gluten‐free bread formulation (based on rice flour (50%) and potato starch (50%)) has also been documented to increase calcium absorption, reduce glycemic index (from 71 to 48), and glycemic load (from 12 to 8) (Capriles & Arêas, 2013). The use of 100 g inulin in the diet of heterogeneous rats with human flora is also associated with increased sulfomucin production and decreased sialomucin (indicating a less risk of colon cancer) (Fontaine et al., 1996).

Since the health benefits of inulin require adequate consumption, including inulin in bread may be a good strategy in a cereal‐based diet to ensure adequate intake. However, the technology performance of this combination is also crucial. Considering the importance of the technological properties of bread for consumer acceptance, the following section examines the influences of inulin addition on these properties.

## 3 TECHNOLOGICAL CHARACTERISTICS OF BREAD CONTAINING INULIN

Technological characteristics are effective parameters in predicting the mixing behavior of the dough, fermentation characteristics, baking efficiency of food production, and textural characteristics of the final product. The textural, rheological, and sensory characteristics of bread are the most important technological characteristics that determine the final product's quality and the consumer's acceptance (Morris & Morris, 2012). In this regard, water absorption of bread formulation can significantly affect its technological characteristics by influencing the above parameters. Consequently, an increase in water absorption can improve the specific volume properties, dough expansion (Han et al., 2012), and the formation of large cavities in the bread crumb (the bread crumb is a white porous structure located under the crust, while the crust is the brown surface layer of the bread) (Mohammadi et al., 2021), as well as the reduction in the hardness and staling rate of the bread (Morris & Morris, 2012). Furthermore, the amount of water absorption also determines the color of the bread crust, with low humidity promoting the Millard reaction and creating a darker color (Mohammadi et al., 2021).

It is worth mentioning that the incorporation of inulin into bread formulation could improve its technological properties due to its high capacity to interact with water (Hager et al., 2011). Moreover, it can prevent the formation and recrystallization of ice crystals and avoid damaging the proteins during the dough‐freezing process. Additionally, the presence of hydrogen groups in the inulin structure and the hydrophobic reactions between inulin and proteins can have a protective effect on the gluten network. For instance, it was reported that frozen dough containing 2.5% long‐chain inulin had a greater protective effect on gluten protein compared with that containing 5% short‐chain inulin. This phenomenon can be explained by the formation of denser gluten structures by the former type of inulin. Long‐chain inulin is also used in gluten‐free bread as a substitute for gluten (due to the formation of the gel structure) which could improve the technological properties of the final bread (Selmo & Salas‐Mellado, 2014). Several parameters, including the presence or absence of gluten and the strength of the gluten network, influence the effects of inulin on the main technological properties of wheat bread, such as water absorption, special volume, texture, color, and staling, which will be discussed in detail in the following sections.

### 3.1 Water absorption

Water is one of the main components in the formation and stability of dough. Water absorption is defined as the ratio of water volume to flour weight required to produce dough with standard consistency (Belz et al., 2012). The quality and shelf life of products are strongly influenced by the moisture distribution and the interaction of water with other compounds in the dough formulation (Luo et al., 2017). In this regard, as the baking temperature of the bread increases, the water acts as an insulator, preventing the evaporation of moisture from the bread crumb. Furthermore, water absorption of the dough and the resulting moisture can directly affect the texture, volume, color, and stale properties of bread (Morris & Morris, 2012).

Since inulin hydrocolloids have abundant hydroxyl groups in their structure and form gel‐like structures, they may play an important role in water absorption by maintaining and increasing it (Luo et al., 2017). Factors such as the amount and strength of protein, the type and content of starch, the presence of dietary fiber in the formulation, DP, and the level of inulin replacement affect the performance and behavior of inulin concerning water absorption by influencing gel formation (Bojnanska et al., 2015; Ziobro et al., 2016). Additionally, adding gluten protein to the dough increases its water absorption, stability time, and strength (Fan et al., 2021). Considering the effect of protein strength on dough water absorption, Shiri et al. (2021) revealed that an enhanced replacement level of acorn flour compared to rice flour in gluten‐free bread containing 10% w/w long‐chain inulin decreased the water absorption capacity. This can be explained by the lower amount of acorn flour protein (5.40 wt%) and the potential effect of protein on water retention (Shiri et al., 2021). Similarly, Hrusková et al. (2019) found that the dough's water absorption decreased with a higher proportion of chestnut flour in white wheat bread due to the high increase in sucrose and the inhibitory effect of gel formation by chestnut flour (Hrusková et al., 2019).

Table 1 presents the effect of inulin with different properties on water absorption of different pieces of bread investigated in previous studies. According to the previous research, the addition of inulin reduces the water absorption and moisture of the dough, as inulin creates a structure with low elasticity and high viscosity, by weakening the gluten network and at the same time forming areas of high water inclusion capacity (Ahmed et al., 2020; Mohammadi et al., 2021). Therefore, less water is needed to achieve a dough with the desired consistency. It should be noted that short‐chain inulin had a greater effect on reducing dough water absorption compared with long‐chain inulin in constant water content. This can be a result of the fact that low DP inulin contains simple mono‐ and oligosaccharide sugars that compete with starch granules for water absorption, which delays the gelatinization of starch and reduces the dough water absorption. However, long‐chain inulin was not significantly different from the control sample in terms of water absorption. This could be due to the lower solubility and ability to form a gel‐like structure of the longer chain (Ziobro et al., 2013). Additionally, the powder form of inulin reduces the water absorption of the dough more than the gel form due to its low water solubility. However, gels have a higher rate of interaction with water (Chiavaro et al., 2007; Morris & Morris, 2012). As a consequence, it can be argued that inulin contributes crucially to the stability and structure of the final dough by influencing water absorption.

**TABLE 1:** Effect of inulin properties on water absorption in different breads

Inulin properties	Type of bread	Inulin substitution level (w/w %)	Water absorption ratio (%)	Moisture content (%)	Results	Reference
Short‐chain (S, DP ≤ 10), Native (N, DP2 ~ 60), Long‐chain (L, DP ≥ 23	Plain wheat bread	5 (S) 5 (N) 2.5 (L)	56.76 (S) 56.01 (N) 60.98 (L)	38.03 (S) 38.15 (N) 39.35 (L)	Water absorption and moisture content were reduced with the substitution of 5% of FS and 5% of FI, but 2.5% of FXL was not significantly different from the control sample.	Luo et al. (2017)
Short‐chain (S, DP ≤ 10), Native (N, DP2 ~ 60), Long‐chain (L, DP ≥ 23	Wheat bread	10	40.24 (S) 39.60 (N) 39.60 (L)	–	The amount of freezable water was reduced with the addition of three types of inulin compared to the control sample.	Luo et al. (2017)
Short‐chain (S, DP ≤ 10), Native (N, DP2 ~ 60), Long‐chain (L, DP ≥ 23	Gluten‐free bread	12	59 (S) 59 (N) 65 (L)		Water absorption of the dough was reduced with the addition of short‐chain and natural inulin, but long‐chain inulin increased water absorption.	Ziobro et al. (2013)
Inulin gel: Low‐sugar inulin, DP ⩾ 8 Inulin powder: Low‐sugar inulin, DP ⩾ 8	Wheat bread	2.5	59.5 (Inulin gel) 56.3 (Inulin powder)	–	Water absorption of the dough decreased with the addition of inulin in powder form. In contrast, the addition of gel‐form inulin increased the water absorption of the dough compared to the control sample.	O'brien et al. (2003)
Short‐chain (S, DP ≤ 10), Native (N, DP2 ~ 60), Long‐chain (L, DP ≥ 23)	Wheat bread	10	53.71 (S) 57.52 (N) 62.01 (L)	35,00 (S) 31.07 (N) 25.94 (L)	Water absorption and moisture decreased with the addition of inulin	Mohammadi et al. (2021)
Long‐chain inulin	Gluten‐free bread (Acorn flour 10% and 90% Rice flour) Gluten‐free bread (Acorn flour 30%:70% Rice flour) Gluten‐free bread (Acorn flour 50%:50% Rice flour)	10	73 (Acorn: Rice 10:90) 64 (Acorn: Rice 30:70) 63 (Acorn: Rice 50:50)		Water absorption and moisture absorption reduced with the replacement level of acorn flour increased.	Shiri et al. (2021)
Chicory‐derived inulin	Arabic bread (brown wheat flour)	1.25, 2.5 3.75	63.1, 59.9, and 57.5, respectively (ascending ratio)	–	The water absorption of the dough was significantly reduced by replacing 3.75% inulin.	Ahmed et al. (2020)
Inulin from chicory	Wheat bread	5, 10, 15, 20, and 25	56.2, 57.1, 58.5, 60.3, and 61.8, respectively	–	The water absorption of the dough decreased with the substitution level of lower inulin (5 and 10). However, replacing higher levels of inulin (15%–25%) increased the dough water absorption compared to the control sample.	Bojnanska et al. (2015)

### 3.2 Specific volume

The specific volume index determines the ability of the dough to retain and develop the carbon dioxide produced during the fermentation and baking process (Nunes et al., 2009). It is affected by factors such as water absorption rate, the intensity of the baking process, the performance of the fermentation process, and the aeration rate during kneading (Carr & Tadini, 2003).

The addition of inulin, as one of the factors affecting the specific volume, is influenced by several factors, including the type of dough matrix (presence or absence of gluten and gluten network strength), type of fermentation process (*Saccharomyces cerevisiae* yeast and sourdough), amount, DP, and form of inulin (powder or dehydrating [gel]). Generally, the inclusion of inulin in the dough may alter the formation of disulfide bonds and retention of CO2 gases by delaying the gelatinization of starch and reducing water absorption (Ziobro et al., 2013). It is revealed that the addition of inulin to gluten‐free bread improves several features such as viscosity, gas storage capacity during the baking process, and specific volume. These positive effects are attributed to its hydrophilic properties and the formation of a gel‐like structure. However, inulin addition to gluten‐containing bread reduces the specific volume compared with the control sample due to a reduction in the elasticity of the dough and weakening of the gluten network (Kou et al., 2019). There have been inconsistent results in previous studies, which were discussed below.

Regarding inulin DP, it is reported that short‐chain inulin cannot form a gel; natural inulin forms a gel in concentrations above 30% w/w. In contrast, long‐chain inulin forms a gel in the range of 20%–40% w/w at room temperature due to its lower solubility in water. Consequently, it positively affects the increase in the specific volume (Chiavaro et al., 2007). Likewise, Morris and Morris (2012) revealed that compounds containing long‐chain inulin in gluten‐containing bread had a higher specific volume than short‐chain types which is due to the formation of a gel‐like structure and compensation for the weakening of the gluten network. However, short‐chain inulin in gluten‐free bread is easily hydrolyzed by yeasts due to the presence of simple monosugars and oligosaccharides, increasing gas production and thus increasing specific volume (Ziobro et al., 2013). As observed in a study by Ziobro et al. (2013), by adding short‐ and medium‐chain inulin (DP < 10 and DP ≥ 10) to gluten‐free bread (based on corn starch and potato starch), the specific volume increased by 25%–33% and 24%–59%, respectively, compared with the control sample. In contrast, the sample containing long‐chain inulin did not differ significantly from the control sample.

Considering the type of fermentation, it has been reported that fermentation with sourdough improves the specific volume resulting from the synergistic effect of bacterial lactic acid on the metabolic activity of *Saccharomyces cerevisiae* and the further release of carbon dioxide. In addition, inulin can partially compensate for the weakening of the gluten network caused by this type of fermentation by producing exopolysaccharides (Katina et al., 2006). Mohammadi et al. (2021) explored that the specific volume of fermented wheat bread with mixed fermentation based on sourdough was significantly higher than samples fermented with *Saccharomyces cerevisiae*. Also, wheat bread containing long inulin fermented chains with sourdough showed a higher specific volume compared with short types of chains fermented with yeast. In this regard, the exopolysaccharides produced during the fermentation of sourdough and fermented long‐chain inulin can synergistically compensate the decrease in the gluten content, due to the addition of inulin level (Kim et al., 2001). Gluten‐free bread fermented with yeast with normal invertase activity (Y_1_) has a higher specific volume than bread fermented with yeast with low invertase activity (Y_2_) due to higher enzymatic activity and gas production (Morreale et al., 2019). As for the effect of inulin addition form on the specific volume, the results of O'brien et al. (2003) showed that inulin inclusion in wheat bread as a gel (containing 13.2% protein) led to a higher specific volume than the powdered state because of increased water absorption (O'brien et al., 2003). Also, increasing inulin replacement as one of the factors affecting specific volume showed that with the increase in inulin substitution (from 5% to 10% w/w) in Chinese steamed bread (CSB), the specific volume decreased to 7.66%. This can be attributed to the decrease in dough elasticity due to the weakening of the gluten network (Liu, Luo, et al., 2016).

### 3.3 Texture

The texture is one of the most important parameters in bakery products, which significantly affects consumer acceptance. Especially for elderly people and those with swallowing and chewing disorders, the texture of food is important. Therefore, the right texture plays an important role in product superiority (Guimarães et al., 2020). The mechanical properties of bread crumbs' texture are determined by testing their properties with a texture profile analyzer (TPA). This involves compressing the sample in two steps, which is similar to the chewing test. The bread texture properties such as hardness (the amount of force required to achieve a certain deformation, sometimes called firmness), cohesiveness (an indicator of the strength of the internal bands that make up the body of the product), chewiness (the product of three indicators of cohesion × elasticity × hardness), elasticity (the rate of return of a material after removal of the force), and adhesiveness (the ratio between the active work done under the second force–displacement curve) are important characteristics of the bread quality that could be affected by inulin addition to the bread formulation (Guimarães et al., 2020). The effect of inulin on these properties can be influenced by factors affecting inulin function including polymerization degree and inulin replacement level, type of fermentation, protein strength, and so on (Morris & Morris, 2012). Table 2 presents the effect of inulin addition on the textural properties of different bread.

**TABLE 2:** Effect of different inulin addition on textural properties of different breads.

Type of bread	Inulin properties	Matrix properties	Parameters				Results	References
			Firmness	Cohesiveness	Elasticity (mm)	Chewiness (mJ)		
			(N)					
Steamed bread/plain wheat flour	Short‐chain (S, DP ≤ 10), Native (N, DP2 ~ 60), Long‐chain (L, DP ≥ 23	Inulin addition at 5, 5, and 2.5% w/w for S, N, and L, respectively	11.02 (S)	0.66 (S)	0.89 (S)	6.48 (S)	The initial firmness and chewiness of steamed bread increased with the substitution of FI 5%, FI 5%, and FXL 2.5%, but cohesiveness and elasticity decreased. The effect of FXL was significant.	Luo et al. (2017)
			16.3 (N)	0.63 (N)	0.86 (N)	8.74 (N) 8.98 (L)		
			18.29 (L)	0.63 (L)	0.83 (L)			
Wheat bread	Long‐chain inulin (L, DP = 23)	Flour W (weak)	73.6 (W, L)	–	–	–	Long‐chain inulin had adverse effects on the hardness of bread made with weak and strong flour.	Brasil et al. (2011) and Peressini and Sensidoni (2009)
	Short‐chain inulin (S, DP = 10)	Flour MS (moderately strong)	32.5 (MS, L)					
			27.2(W, S)					
			9.2 (MS, S)					
Chinese steamed bread	Short‐chain inulin (DP ≤ 10)	Inulin addition at 5%, 7%, and 10% w/w	3.53, 10.24, and 8.59 N for 5, 7 and 10% w/w inulin‐containing samples	0.71, 0.58, and 0.57 for 5, 7, and 10% w/w inulin‐containing samples, respectively	0.89, 0.81, and 0.76 mm for 5, 7, and 10% w/w inulin‐containing samples, respectively	2.97, 4.82, and 3.70 mJ for 5, 7, and 10% w/w inulin‐containing samples, respectively	All textured parameters of steamed bread were improved by replacing 5% short‐chain inulin, and the hardness was reduced by 65.86% compared to the control sample when 5.0% inulin was added	Liu, Luo, et al. (2016)
Steamed bread (low‐gluten flour)	Short‐chain (S, DP ≤ 10), Native (N, DP2 ~ 60), Long‐chain (L, DP ≥ 23	Inulin added (5%)	3.64 (S)	0.81 (S)	0.93 (S)	2.74 (S)	The addition of FS (5%) and FI (5%) improved the textural properties of steamed bread made from soft flour, but the addition of FXL had a negative effect.	Kou et al. (2019)
			3.81 (N)	0.82 (N)	0.91 (N)	2.82 (N)		
			12.7 (L)	0.74 (L)	0.91 (L)	8.45 (L)		
Gluten‐free bread (rice flour)	Inulin mix of oligo‐ and polysaccharides, DP 2–20	Yeast with normal (Y1) invertase activity	2.20 (Y1)	0.76 (Y1)	2.15 (Y1)	248 (Y1)	The fermented bread with yeast (Y2) had higher stability of inulin and lower hardness than yeast (Y1).	Morreale et al. (2019)
		Yeast with reduced (Y2) invertase activity	1.42 (Y2)	0.80 (Y2)	5.49 (Y2)	554 (Y2)		
Wheat bread	Short‐chain (S, DP ≤ 10), Native (N, DP2 ~ 60), Long‐chain (L, DP ≥ 23	Common yeast	35.09 (S), 33.26 (N), 31.11 (L)		8.9 (S), 9.04 (N), and 9.61 (L)	147 (S), 118 (N), and 158 (L)	The hardness decreased with increasing degree of polymerization	Mohammadi et al. (2021)
Gluten‐free bread (rice and acorn flour)	Long‐chain inulin (DP ≥ 23)	1‐Acorn flour 10%:90% Rice flour	75.67 (1)	0.28 (1), 0.40 (2), 0.24 (3)	11.50 (1), 12.50 (2), 7.40 (3)	65.72 (1), 40.92 (2), 180.74 (3)	The hardness increased, and the cohesiveness decreased with increasing the level of acorn flour (50%) (reducing the amount of protein)	Shiri et al. (2021)
		2‐Acorn flour 30%:70% Rice flour	40.97 (2)					
		3‐Acorn flour 50%:50% Rice flour	131.0 (3)					
Arabic bread	Chicory‐derived inulin	Inulin addition at 1.25%, 2.5%, and 3.75%, respectively	20.09, 21.09, and 24.06 N for inulin addition at 1.25%, 2.5%, and 3.75%, respectively	0.01, 0.02, and 0.03 for inulin addition at 1.25%, 2.5%, and 3.75%, respectively			The hardness and cohesiveness increased with increasing the level of inulin replacement	Ahmed et al. (2020)

As the most important indicator of textural properties, hardness is directly related to chewiness (Liu, Mu, et al., 2016). It was reported that the hardness of wheat bread samples increased with the addition of inulin, which is related to the DP of inulin (Morris & Morris, 2012). Overall, the hardness of wheat bread kernels increased with the addition of inulin because the elasticity of the dough decreased, the gluten network was weakened, and thus the gas storage capacity was reduced. The hardness degree is also affected by the type of fermentation. For example, wheat bread samples fermented by mixed fermentation based on sourdough had significantly higher hardness and chewiness than samples fermented by *Saccharomyces cerevisiae*. A possible explanation for this could be increased proteolytic activity in the mixed fermentation based on sourdough. Therefore, hydrolysis of various gluten residues results in a system with less flexibility and elasticity (Fekri et al., 2020). Cohesiveness is an indicator of the internal bands' strength of the product. Higher cohesion means a better ability to maintain the integrated structure of the bread, which tends to decrease with increasing inulin content in wheat bread due to the reduction in disulfide bonds and the weakening of the gluten network.

### 3.4 Color

Color is an important quality characteristic of bread, which is measured with a colorimeter. This characteristic can be determined by the following parameters: brightness index (L*) varying between 0 (black) and 100 (white), as well as the blue–yellow index (b*) and green–red index (a*), which range from −127 to +127 (Moghaddasi et al., 2020).

Inulin, as one of the effective factors for bread color, increases the rate of the Maillard reaction, the formation of brown nitrogen polymers, and melanoidin during baking. Hence, inulin addition can improve the color of the bread crust (especially in gluten‐free bread, which usually has a weak color compared to wheat bread) (Drabińska et al., 2016). It is noticeable that the color of the bread crust mainly results from the Millard reaction products, while the bread crust color is mainly influenced by the components of the formulation (Conforti & Davis, 2006). In this context, Mohammadi et al. (2021) showed that the crumb color of wheat bread enriched with inulin fermented by mixed fermentation based on sourdough had the highest specific volume and the lowest brightness. This is because the increase in enzymatic activity by the sourdough microorganisms leads to a more porous texture and greater light scattering, thus reducing brightness (L*). Considering the effect of inulin replacement level as one of the practical factors in creating crust color, Liu, Luo et al. (2016) found that the replacement of 5% inulin increased the L* values of CSB crust compared with the control sample. On the other hand, inulin is whiter than flour. Also, Inulin can bind with starch, protein, and water to create a uniform structure that reflects light. Therefore, this structure produces a whiter color when light strikes the uniform surface of the bread (Liu, Luo, et al., 2016).

Considering the effect of the yeast type used during the fermentation process on the amount of color produced, Morreale et al. (2019) identified that gluten‐free bread fermented with yeast Y_1_ showed greater a* and b* values compared with gluten‐free bread fermented with yeast Y_2_ because yeast Y_1_ can increase free sugar production and Millard reaction rate compared to Y_2_ (Morreale et al., 2019). Similarly, Mohammadi et al. (2021) explored that wheat bread samples containing short‐chain inulin (DP < 10) fermented with sourdough had lower L* and higher a* and b* than samples containing long‐chain inulin fermented with *Saccharomyces cerevisiae* yeast. Since crust color is mainly due to the Millard reaction, samples with short‐chain inulin fermented with sourdough are more susceptible to the Millard reaction than samples with long‐chain inulin fermented with *Saccharomyces cerevisiae* due to the more simple sugars and increased enzymatic activity. Overall, the bread color containing inulin is also affected by the DP of inulin. In this regard, Peressini and Sensidoni (2009) showed that the sample containing short‐chain inulin had a higher Millard reaction rate and, consequently, a browner crust color than the sample containing long‐chain inulin. This can be attributed to the presence of more reducing sugars in the former type (Peressini & Sensidoni, 2009). It should be noted that the state of the added inulin has no significant effect on color. In this regard, Hager et al. (2011) reported that the color of the crust was the same for all levels (2.5%, 5%, and 7.5%) of added inulin in two forms (gel and powder) (Hager et al., 2011).

### 3.5 Staling

The staling process occurs when hydrophilic compounds of flour migrate from the crust to the crumb (Peressini & Sensidoni, 2009) as a result of the interaction between hydrophilic flour compounds (gluten‐starch) and retrograded starches (Ziobro et al., 2013). The bread staling occurs through the accumulation of amylopectin in the starch structure. Baking, on the other hand, causes the starch to gelatinize, and the amylase to separate from the amylopectin during the process. During cooling, gelatinized starch changes from amorphous to crystalline (retrogradation). Thus, the linear amylose molecules and the linear part of amylopectin can be categorized as rigid crystal structures (Drabińska et al., 2016). In general, the staling process is associated with a decrease in bread quality as well as an increase in hardness. This process leads to adverse changes in the appearance, taste, and texture of the product, which lowers customer acceptance and increases waste (Ziobro et al., 2013). However, white bread is one of the most important diet components but has the shortest commercial shelf life among processed food products due to its rapid staling (Ziobro et al., 2013).

**FIGURE 2:**
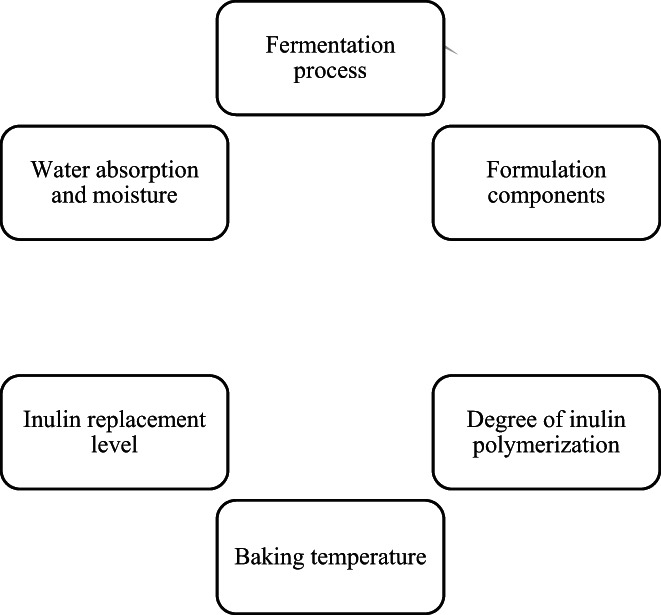
Factors affecting the performance of inulin on bread staling

Inulin inoculation, as one of the effective factors impacting the staling rate, causes the partial distribution of water. It can bind to starch through hydrogen bonds and change the water distribution between starch and protein in gluten‐containing bread (Kou et al., 2019). Furthermore, inulin, as a hydrocolloid substance with high water‐holding capacity, could reduce water loss during bread storage (Hager et al., 2011). In other words, inulin prevents amylose leaching during baking by forming a barrier around starch granules, which affects the staling rate in the first hours of storage (Maga & Ponte, 1975; Ziobro et al., 2013). Factors affecting inulin performance on bread staling are shown in Figure 2.

The effect of inulin on the staling rate of gluten‐free and gluten‐containing bread depends on several factors, including inulin characteristics such as DP, extraction source, and addition method (Maga & Ponte, 1975; Ziobro et al., 2013). In this regard, the study results of Drabińska et al. (2016) showed that the staling rate of gluten‐free bread decreased by adding 5% inulin compared to the control bread sample (Drabińska et al., 2016). By contrast, Hager et al. (2011) found an increase in staling rate with the addition of 5% inulin, probably due to differences in inulin extraction sources (Hager et al., 2011). Similarly, Ziobro et al. (2013) study documented that with the increasing DP inulin, the hardness and staling rate of gluten‐free bread increased. This phenomenon can be attributed to the reduction in specific volume (due to lower gas production) by the use of long‐chain inulin (DP > 23) (Ziobro et al., 2013). This outcome is contrary to that of (Kim & D'appolonia, 1977), who observed that as DP increased, the staling rate decreased due to the formation of gel structure and less moisture loss.

As for the effect of fermentation type on staleness, Morreale et al. (2019) revealed that gluten‐free bread fermented with Y_1_ yeast was harder than gluten‐free bread fermented with Y_2_ (with lower invertase activity). This may be attributed to the reduced stability of inulin (Morreale et al., 2019). O'brien et al. (2003) also investigated the effect of added inulin type on staling rate. Inulin in powder form increased the hardness of wheat bread, while inulin in gel form did not affect this characteristic of bread staling (O'brien et al., 2003). Bread storage time had a significant effect on inulin performance in changing the stale rate, as a study by Kou et al. (2019) showed that inulin increased the stale rate of soft flour bread (12.9% moisture, 9.7% protein, 0.47% ash, and 22.2% gluten protein) in a short time (less than a day) after baking. The staling rate of bread decreased over a long period (more than a day) because inulin increases starch crystallization during short‐term storage. Additionally, another possible reason could be its high ability to enclose water and decrease the migration of water from the crumb to the crust (Kou et al., 2019). Likewise, Capriles and Arêas (2013) observed similar results by investigating that gluten‐free bread fortified with inulin in the first hours (2, 24, and 48) after baking was firmer than the control bread. However, over long‐term storage, inulin‐containing bread had a softer texture caused by the redistribution of water between components with a lower staling rate than control bread (Capriles & Arêas, 2013). Overall, it can be argued that the staling rate of gluten‐free and gluten‐containing bread decreased with the increasing amount of inulin addition, regardless of its type. This is because inulin contains a large number of hydrophilic groups that slow down the migration of water from the breadcrumbs to the crust.

## 4 FACTORS AFFECTING THE STABILITY OF INULIN DURING THE BREAD PROCESSING STAGE

**FIGURE 3:**
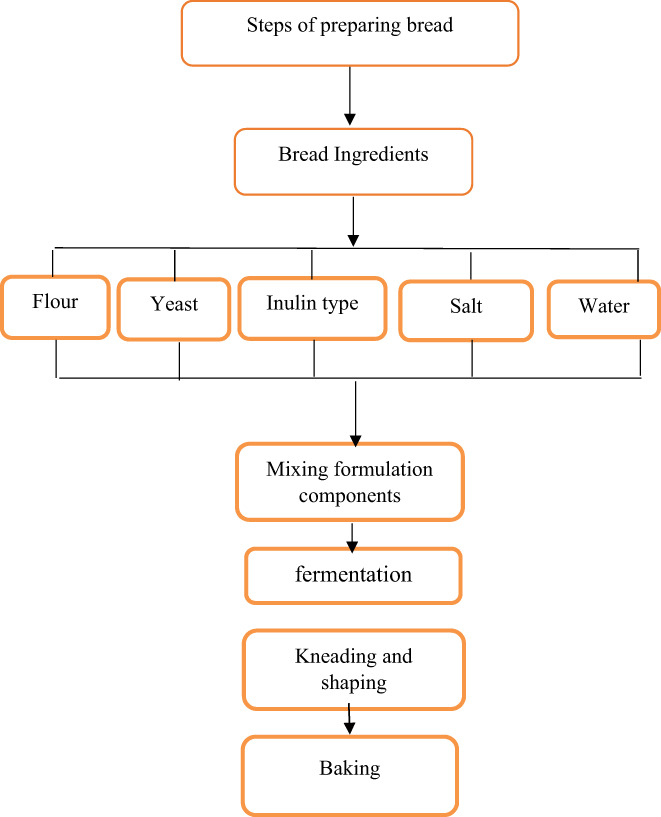
Factors affecting the stability of inulin during the preparation of bread

The lack of dietary fiber in most bread and the worldwide importance of wheat bread as a staple food make inulin fortification an excellent strategy for improving bread quality and human health. Despite the resistance of β‐configuration of inulin to the hydrolytic activity of upper gastrointestinal enzymes, its stability may be affected by the formulation components, pH and acidity of the fermentation process, and baking temperature during the processing step (Mohammadi et al., 2021). Therefore, the most important factors affecting inulin stability were discussed in this study. Figure 3 illustrates the factors affecting the inulin stability during the bread preparation process.

### 4.1 Formulation components

The main ingredients in bread are flour, water, salt, and yeast which are consumed after fermentation and baking (El Khoury et al., 2018). There is about 40% free water in the dough (Belz et al., 2012). Water absorption is one of the most important parameters determining the stability of inulin. It depends on the protein content, the presence or absence of gluten, the degree of starch damage (starch content), and the amount of inulin added and its properties. Also, water absorption is effective in determining the thermal sensitivity of inulin during the baking process. Belz et al. (2012) identified that increasing the amount of free water at temperatures above 135°C causes thermal degradation of long‐chain polysaccharides (Belz et al., 2012). As the most important component of flour, protein effectively increases the quality of the final product and the stability of inulin by improving the water absorption power, the viscosity of the dough, and the ability to retain the fermented gas (Kadan et al., 2001). Water absorption and inulin retention are also influenced by the source and extent of starch damage, as well as the quantity and quality of protein and flour particle size (the smaller the particle size, the more water is absorbed and the firmer the dough). Approximately 80% of wheat bread is starch, which plays a crucial role in its structure (El Khoury et al., 2018). Starch is mainly linear, but amylopectin, which is branched (1–6), causes swelling or water absorption. Hence, starches with a high concentration of amylopectin will absorb more water compared with amylose. Furthermore, damaged starch absorbs more water than intact one; consequently, it can affect the inulin stability (El Khoury et al., 2018). Since the increase in pH affects the stability of inulin, the addition of salt together with the strengthening of the gluten network (Pashaei et al., 2021) can reduce the water activity and the activity of microorganisms in the dough pH, especially yeast and lactic acid bacteria, and increase the stability of inulin. In addition, salt reduction may increase yeast activity, which may lead to increased consumption of free sugars and decreased stability of inulin. Therefore, all of the above may affect the stability of inulin during processing (Hutkins, 2008).

### 4.2 Fermentation process

One of the stages of bread preparation is the leavening of the dough, which can be performed by fermentation and mechanical or chemical agents (Hui & Evranuz, 2012). During bread preparation, fermentation is not meant to ensure longer shelf life, but to convert cereal flour into a usable and consumable product (Rollán et al., 2010). In other words, during the fermentation, yeasts in the dough use carbohydrates to produce carbon dioxide and alcohol and synthesize acids and aromatics that improve the taste, aroma, and shelf life of the bread (Morreale et al., 2019). Generally, the fermentation process can be carried out by two methods: *Saccharomyces cerevisiae* yeast and fermentation with sourdough. Only *Saccharomyces cerevisiae* yeast is used in the yeast fermentation process, but sourdough is a mixture of water and flour fermented by lactic acid bacteria and yeasts (Mohammadi et al., 2021). In comparison, yeast‐based bread has a pH of about 5–6, while sourdough bread has about 4 because of the lactic acid bacteria it contains (Hutkins, 2008). Various fermentation processes can alter the stability of inulin by affecting the pH and thus the optimal conditions for enzyme activity.

Despite several benefits of fermentation, previous studies have shown the destructive effects of fermentation on inulin stability. Mohammadi et al. (2021) documented that inulin stability was lower in sourdough fermentation than in yeast due to longer fermentation time and higher enzymatic activity (Mohammadi et al., 2021). Also, because the yeast invertase enzyme is effective in degrading inulin‐type fructans, it was reported that gluten‐free bread fermented with yeast with low invertase activity had higher stability of inulin than gluten‐free bread fermented with yeast with normal yeast invertase activity (Hutkins, 2008). Moreover, the study results by Glibowski and Bukowska (2011) reported that the stability of inulin in acidic products (pH ≤ 4) decreased at temperatures above 60°C, while in products with a pH ≥5, there was no inulin degradation even at 100°C (Glibowski & Bukowska, 2011). It has also been demonstrated that the prebiotic activity of inulin is stable for 24 h at low pH (3–6) (Glibowski & Wasko, 2008). However, when it was heat treated (85°C) for 30 min, its prebiotic activity decreased (Huebner et al., 2008). Since heating at low pH is associated with a decrease in prebiotic activity, inulin addition to acidic products is limited, especially when the process temperature exceeds 60°C.

### 4.3 Baking process

Baking bread is a physical, biochemical, and microbiological process that involves putting the sticky, spongy, and inedible dough in the oven and creating a combination with a unique flavor or bread (Mondal et al., 2008). The most apparent changes in this period are volume expansion, crust formation, inactivation of yeasts and enzymes, protein coagulation, partial gelatinization of starch, and moisture loss (Belz et al., 2012). Baking time and temperature affect the stability of inulin; at the same baking temperature, short‐chain inulin is more vulnerable than long‐chain inulin (Glibowski & Wasko, 2008). The study by Mohammadi et al. (2021) reported that inulin loss during baking in the formulation containing short‐chain inulin fermented with mixed sourdough‐based fermentation was 41%; however, when treated with long‐chain inulin and fermented with *Saccharomyces cerevisiae* yeast, this value was about 25%. This lower percentage may be attributed to the lower solubility of long‐chain inulin and the lower enzymatic activity of *Saccharomyces cerevisiae*. Although the chemical structure of inulin changes during the heating process, its functional activity may remain the same or may even increase after processing. As reported in the study by Bohm et al. (2006), dry heat (135–195°C) for 30 min leads to the destruction of 20%–100% of fructan chains and the production of new low‐molecular‐weight products (Di‐D‐fructose dianhydrides). Moreover, its addition to the fecal culture medium significantly stimulated the growth of Bifidobacterium and Enterobacteriaceae and reduced considerably the growth of potential pathogens.

One of the reactions that occur on a large scale during bread baking is the Millard reaction, which is a nonenzymatic reaction between amino acids and reducing sugars at temperatures above 110°C. During this mechanism, increasing the surface temperature of the product causes the evaporation of unbonded water (free water), decreases the moisture content, reduces the water activity of the crust, and increases the Millard reaction rate (Huebner et al., 2008). Furthermore, factors affecting the decrease in pH (including the type of fermentation and the browning reaction associated with the loss of the amino group) can influence the Millard reaction rate (Hutkins, 2008). A possible explanation for the effect of inulin on the Millard reaction is reducing sugars, which accelerates the Millard reaction by decreasing the amount of free water. The Millard reaction may be favored when low‐molecular‐weight fructans are hydrolyzed during processing, thus increasing the amount of free sugar (especially fructose). Thereby, the Millard reaction is accelerated by inulin in formulations. Millard reaction can also reduce the prebiotic activity of carbohydrates. Several studies have demonstrated that when inulin is converted into short‐chain oligosaccharides or monosaccharides, its prebiotic function diminishes and brown color is formed during the reaction (Huebner et al., 2008).

## 5 CONCLUSION

Overall, adding inulin to bread improved its technological properties. The purpose of this study was to examine the technological characteristics of bread enriched with inulin and to analyze factors such as the DP of inulin and the inulin type that affect the stability of inulin to improve its nutritional performance and health benefits. The effects of inulin replacement in bread formulation were analyzed such as textural and color, specific volume, water absorption, and staling pointed. In addition, several vital factors in the enhancement of these effects were studied, including the type of bread, the DP of inulin, and the type of fermentation. Inulin‐type fructans reduce the staling rate and maintain the freshness of gluten‐free and gluten‐containing bread due to their hydrophilic properties. Furthermore, the effect of formulation components, fermentation, and baking process on the stability of inulin revealed that short‐chain inulin had less stability than long‐chain inulin. This could be attributed to the presence of simple sugars (which are more accessible to yeasts) as well as low pH. On the other side, due to the high content of reducing sugars in the structure of inulin, short‐chain inulin increases the Millard reaction rate and forms a more attractive crust color. Overall, it can be concluded that inulin is almost stable under acidic conditions, but is highly susceptible to degradation by heating at low pH levels.

## FUNDING INFORMATION

Fasa University of Medical Sciences supported this study, grant no. 400148.

## CONFLICT OF INTEREST

The authors declare that they have no conflict of interest.

## CONSENT FOR PUBLICATION

Not applicable.

## ETHICS APPROVAL

This study was approved by the Fasa University of Medical Sciences. Approval ID: IR.FUMS.1400.115.

## Data Availability

The datasets used and/or analyzed during the current study are available from the corresponding author on reasonable request.
